# Identification of Three *Dalbergia* Species Based on Differences in Extractive Components

**DOI:** 10.3390/molecules23092163

**Published:** 2018-08-28

**Authors:** Xiaoqian Yin, Anmin Huang, Shifeng Zhang, Ru Liu, Fang Ma

**Affiliations:** 1 Research Institute of Wood Industry, Chinese Academy of Forestry, Beijing 100091, China; yxqwzy@163.com (X.Y.); liuru@criwi.org.cn (R.L.); 2 Beijing Key Laboratory of Wood Science and Engineering, Beijing Forestry University, Beijing 100083, China; shifeng.zhang@bjfu.edu.cn; 3 Department of Chemistry, Tsinghua University, Beijing 100084, China; mf690772309@163.com

**Keywords:** *Dalbergia* spp., distinction, extractives, FTIR, GC-MS

## Abstract

*Dalbergia cultrate*, *Dalbergia latifolia*, and *Dalbergia melanoxylon* are precious and valuable traded timber species of the genus *Dalbergia*. For chemotaxonomical discrimination between these easily confused species, the total extractive content of the three wood species was determined using four different organic solvents. Fourier transform infrared (FTIR) spectroscopy was used to analyze functional group differences in the extractive components, inferring the types of principal chemical components according to characteristic peak positions, intensities, and shapes. Gas chromatography-mass spectrometry (GC-MS) was carried out a detailed characterization of the extractive components. The relative content of individual chemical components was determined by area normalization. Results revealed differences in the chemical components and total and individual extract contents of the three *Dalbergia* species, indicating that FTIR and GC-MS spectroscopy can be applied to identify and discriminate between *Dalbergia cultrate*, *Dalbergia latifolia*, and *Dalbergia* *melanoxylon*.

## 1. Introduction

Wood extractives are non-structural wood molecules that represent a minor fraction in wood, specifically defined as compounds that can be extracted by polar, non-polar, or neutral solvents [1,2]. Wood extractives account for approximately 2% to 5% of wood content, however, relatively high amounts of extractives can be found in some tropical woods, especially chemical extractives that are highly concentrated in heartwood [3,4,5]. Studies have demonstrated that many extractive components exhibit various biological activities and are important in medical applications [6,7]. Wood extractives include an array of compounds, usually aliphatic, terpenoid and phenolic in nature. According to the literature [8], *Dalbergia* spp. are rich in aromatic compounds, however, significant differences have been found in the types and contents of wood extractives, even within the same genus [3].

*Dalbergia* is a genus of trees belonging to the Fabaceae (pea) family, that includes approximately 250 species. All species of *Dalbergia* spp. were listed in the 17th Convention on International Trade in Endangered Species of Wild Fauna and Flora [9]. *Dalbergia cultrate* (Benth.), *Dalbergia latifolia* (Roxb.), and *Dalbergia melanoxylon* (Guill. & Perr.) are high-profile species renowned for their use in high-quality products worldwide [10,11]. These species are used in luxury furniture, artwork, and musical instruments due to their refined colors and excellent hardness and intensity properties [12,13]. Chemical and physical properties are greatly influenced by extractives, with characteristic colors and textures generating extensive market demand and heavy deforestation. Hence, these species are protected by Appendix II of CITES, *D. cultrate* is included in the list of national key preserved wild plants in the People’s Republic of China (first batch). However, any species appearing in Appendix II of CITES are banned from international commercial trade except for those with an import and export license or re-export certificate [14]. Therefore, a more persuasive method for distinguishing closely related species is needed.

Traditional wood anatomy identification methods based on macroscopic and microscopic characteristics have been well-established for several years [15]. However, wood anatomy is too specialized for legal identification, especially when considering leaves, flowers, fruits, and other information to provide the extent of identification required by CITES [16]. By contrast, chemotaxonomical and genetic methods are useful in wood identification [17]. Previous investigations have found chemotaxonomical identification based on the analysis of extractive compounds to be an effective method for distinguishing extremely similar wood species [18,19,20].

To the best of our knowledge, Fourier transform infrared (FTIR) spectroscopy is a fast, simple, non-destructive method and a powerful technology to determine functional groups in the fingerprint region. This approach has been widely used in identification of complex systems such as traditional Chinese medicines and is suitable for analyzing woody materials [21,22]. In recent years, research on infrared spectroscopy in wood extractive studies has grown in popularity [23,24,25]. Additionally, gas chromatography-mass spectrometry (GC-MS) offers clear advantages when analyzing complex mixtures; the combination of an ideal separation technique (GC) with a sensitive identification technique (MS) constitutes a reliable and commonly used method for qualitative and quantitative analysis of compounds [26,27,28]. GC-MS has been widely applied in establishing chromatographic fingerprints for quality evaluation of herbal medicines [29]. It has also been well-established in the characterization and identification of wood extractive compounds [30,31,32].

Little research has been carried out to identify the three similar *Dalbergia* species *Dalbergia cultrate*, *Dalbergia latifolia*, and *Dalbergia melanoxylon* based on chemical taxonomy. This paper examines differences in extractive contents and components using FTIR and GC-MS approaches to discriminate chemotaxonomically between the extractive components in *Dalbergia* spp. Functional group analysis using FTIR spectroscopy and detailed analysis of their components by GC-MS allow one to classify similar species by their chemical nature. These two techniques also facilitate comprehensive analysis of chemical components to support the chemotaxonomical classification of wood. GC-MS data provide a persuasive basis for specific compounds identified by infrared absorption spectra, as the two methods are complementary and valuable in chemotaxonomical identification.

## 2. Results and Discussion

### 2.1. Contents Analysis of Extractives

The extractive contents of the three *Dalbergia* species are listed in Table 1. *Dalbergia cultrate*, *Dalbergia latifolia*, and *Dalbergia melanoxylon* were extracted with four different polar organic solvents, the extractive contents were calculated, and the average value of three experiments was taken. The findings indicated that the non-polar solvent *n*-hexane was not suitable for wood extraction, indicating that the extractable components of these three kinds of wood are mostly polar. After successfully extracting samples with ethanol/water, benzene/ethanol, ethyl acetate respectively, the extract contents were clearly different. Among the benzyl alcohol and ethyl acetate extracts of the three species *Dalbergia cultrate* had the lowest content, whereas the content of *Dalbergia melanoxylon* was highest. With ethanol/water (9:1, *v*/*v*), the content of *Dalbergia cultrate* was slightly higher than that in *Dalbergia latifolia* but clearly lower than in *Dalbergia melanoxylon*. Therefore, *Dalbergia melanoxylon* contained more types of main compounds than *Dalbergia cultrate*, and *Dalbergia latifolia* had the fewest kinds of principle compounds, likely because it contained fewer polar components. In general, the effect of ethanol/water (9:1, *v*/*v*) was better than benzyl alcohol and ethyl acetate, and more components could be obtained. Therefore, to analyze the primary extractive components in *Dalbergia cultrate*, *Dalbergia latifolia*, and *Dalbergia melanoxylon*, ethanol/water (9:1, *v*/*v*) was used as the final solvent in this experiment.

### 2.2. FTIR Analysis

**Figure 1:**
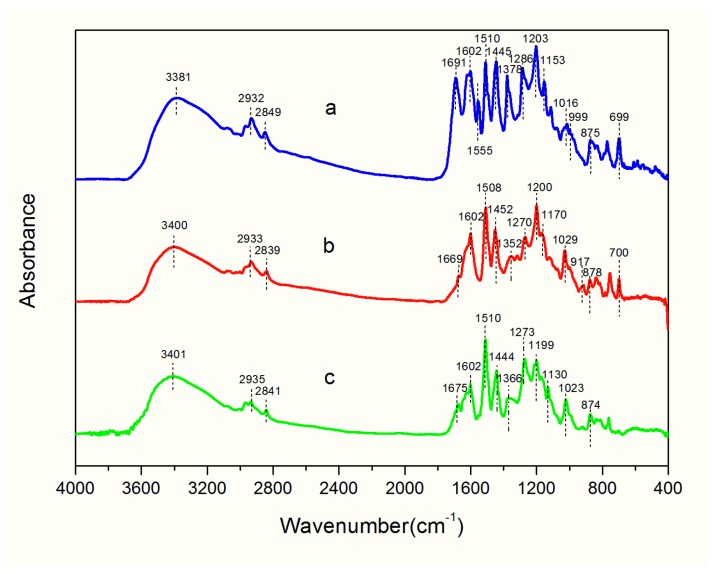
FTIR spectra: (**a**) *D. cultrate*, (**b**) *D. latifolia*, (**c**) *D. melanoxylon.*

The FTIR spectra of the *Dalbergia cultrate*, *Dalbergia latifolia*, and *Dalbergia melanoxylon* extractives are illustrated in Figure 1. Detailed peak positions are summarized in Table 2. The characteristic infrared absorption peaks of the functional groups of the main extractive components were reflected in the FTIR spectra. In the region of 3401–2839 cm^−1^, all spectra exhibited strong, broad peaks attributable to the stretching vibration of O-H or N-H along with a moderate-intensity peak ascribed to methyl and methylene stretching vibrations. Benzene ring characteristic peaks (1602, 1510, and 1450 cm^−1^) of the three species were clear and strong, indicating a large proportion of aromatic substances [33]. However, the relative intensity of these three peaks was distinctly different. Especially for *Dalbergia cultrate*, the intensity of peaks at 1602 cm^−1^ and 1445 cm^−1^ was stronger, suggesting that the number, location, and properties of substituents on aromatic rings varied between the three species. Moreover, *Dalbergia cultrate* showed strong absorption bands around 1691 cm^−1^ for C=O, a peak around 1555 cm^−1^ with medium intensity for C=C of the aromatic ring, and strong absorption peaks at 1286 cm^−1^ and 1153 cm^−1^ for C-O-C [34]; these findings were presumably due to skeletal stretching vibration of the aromatic rings A and B and the functional group C-O-C of ring C of flavonoids [24,35]. These peaks were clearly visible only in *Dalbergia cultrate*; the C=O was weak for *Dalbergia melanoxylon* and was essentially invisible for *Dalbergia latifolia*, indicating an abundance of flavonoid compounds in *Dalbergia cultrate*.

*Dalbergia cultrate* also generated a stronger sharp absorption peak at 1378 cm^−1^. In general, methyl groups have two absorption peaks around 1375 cm^−1^ and 1450 cm^−1^, corresponding to symmetric bending vibrations and asymmetric bending vibrations, respectively [36]; this finding explains why the peak strength of *Dalbergia cultrate* at 1445cm^−1^ was so strong. Then, the absorption peaks at 1366 cm^−1^ of *Dalbergia melanoxylon* and 1352 cm^−1^ of *Dalbergia latifolia* were ascribed to either CH_3_ symmetrical bending or in-plane C-OH bending, as the spectral band shape was low and wide.

Peaks in the region ranging from 910 cm^−1^ to 1300 cm^−1^ were mainly due to C-O single bond stretching vibrations. The extractives of the three species exhibited clear, strong peaks at 1200 cm^−1^ primarily due to C-O-C stretching; however, the band intensity of the three species included typical vibrations around 1270 cm^−1^ (Ar-O) and 1023 cm^−1^ (R-O) for C-O vibrations, although the peak intensity at 1270 cm^−1^ in *Dalbergia melanoxylon* was stronger than that in *Dalbergia latifolia*. *Dalbergia melanoxylon* likely contained more aromatic ether functional groups; a peak appeared at 1130 cm^−1^ for C-OH stretching of the alcohol groups [36]. C-OH stretching near 1114 cm^−1^ was found exclusively in *Dalbergia cultrate*. A C-H out-of-plane bending peak was seen around 999 cm^−1^ in the spectrum of *Dalbergia cultrate* and around 917 cm^−1^ in the spectrum of *Dalbergia latifolia*. Unique vibrations near 700 cm^−1^ were ascribed to the stretching vibration of C-S, which was obvious in *Dalbergia cultrate* and *Dalbergia latifolia*.

### 2.3. GC-MS Analysis

**Figure 2:**
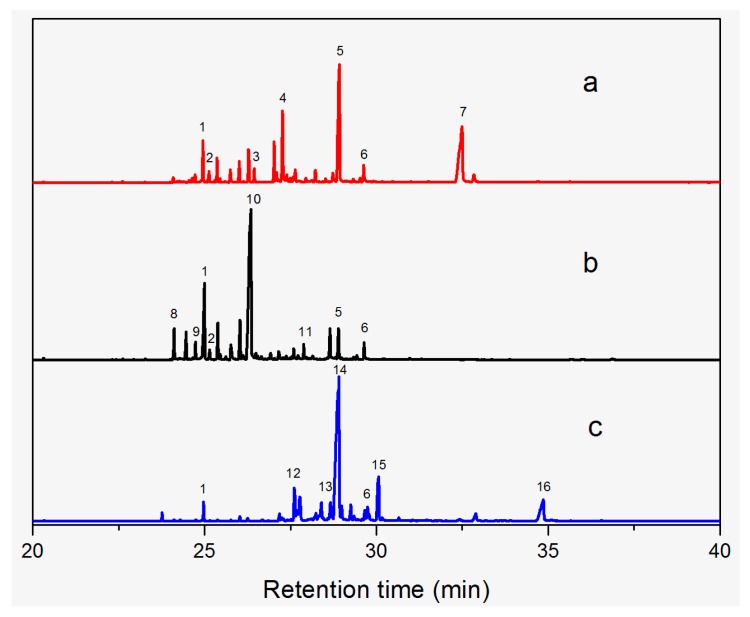
Total ion chromatogram: (**a**) *D. cultrate*, (**b**) *D. latifolia*, (**c**) *D. melanoxylon*.

The GC-MS chromatograms of *Dalbergia cultrata, Dalbergia latifolia,* and *Dalbergia melanoxylon* extractives are presented in Figure 2. The results show clearly the different characteristic peaks of the three species. The peak area was chosen as the analytical signal for the relative amount. The relative content of each chemical component was calculated by area normalization and the average value of the three experiments. Identified chemical components (peak area above 1.0%) and the relative content of these compounds are listed in Table 3. The main chemical components of the three species were determined to be aromatic compounds. In general, these compounds were classified into flavonoids, miscellaneous, quinones, phenols, esters, stilbenoids, and amide compounds. The components of the extracts of *Dalbergia cultrata*, *Dalbergia latifolia,* and *Dalbergia melanoxylon* are detailed below.

Table 3 reveals there were only two common compounds among the three species and each species also contained unique chemical components. The following three compounds were specific to *Dalbergia cultrata*: 3,3′,4,4′-tetramethoxystilbene (peak 3, 1.49%), 3,7,3′,4′-tetrahydroxyflavone (peak 4, 10.78%), and parietin (peak 7, 24.81%). Particular compounds found exclusively in *Dalbergia latifolia* included the following: 1,7,7-trimethyl-3-phenethylidenebicyclo- [2.2.1]heptan-2-one (peak 8, 3.28%), 4,4′-methylenebis-2,6-dimethylphenol (peak 9, 1.60%), 4,2′,3′,4′-tetramethoxy-5′-methyl-6-methylaminomethyl-1,1′-biphenyl (peak 10, 29.75%), (4-methyl- sulfanylphenyl)carbamic acid 2,6-dimethoxyphenyl ester (peak 11, 1.38%). GC-MS analysis of *Dalbergia melanoxylon* revealed five distinct compounds: 10,11-dihydro-10-hydroxy- 2,3-dimethoxydibenz(b,f)oxepin (peak 12, 4.57%), 2-(4-methoxy-2,5-dimethylphenyl)-9-methyl-2*H*- benzo[g]indazole (peak 13, 3.14%), 10,11-dihydro-10-hydroxy-2,3,6-trimethoxydibenz(b,f)oxepin (peak 14, 37.30%), 10,11-dihydro-2,3,6-trimethoxydibenz(b,f)oxepin-10-one (peak 15, 8.32%), and pilloin (peak 16, 7.77%).

According to the GC-MS analysis results, the principal components of the three species varied. Seven constituents were identified as having higher relative contents in *Dalbergia cultrate*: 3,7,3′,4′-tetrahydroxyflavone (peak 4, 10.78%), 7-methoxy-1-thioflavone (peak 5, 25.55%), and parietin (peak 7, 24.81%). 7-Methoxy-1-thioflavone is a flavonoid derivative. Some thioflavones have been reported to function as novel neuroprotective agents and exhibit antiviral activities. 3,7,3′,4′-Tetrahydroxyflavone is a natural flavonol in foods and plants and has been identified as showing various biological activities. Parietin is an anthroquinone and has been identified in the traditional Chinese herbal medicines *Polygoni multiflora* and *Eryngium foetidum* L.

Analysis also revealed the presence of eight compounds in *Dalbergia latifoli* with 4-methyl- 2-[5-(2-thienyl)pyrazol-3-yl]phenol (peak 1, 12.41%) and 4,2′,3′,4′-tetramethoxy-5′-methyl-6-methyl- aminomethyl-1,1′-biphenyl (peak 10, 29.75%) being the most predominant. Both compounds were previously identified in *Dalbergia Stevenson* based on GC-MS results [25,37]. Seven components were recognized in *Dalbergia melanoxylon*, with larger proportions of 10,11-dihydro-10-hydroxy-2,3,6-trimethoxydibenz(b,f)oxepin (peak 12, 37.30%), 10,11-dihydro- 2,3,6-trimethoxydibenz(b,f)oxepin-10-one (peak 14, 8.32%), and pilloin (peak 16, 7.77%). 10,11-Dihydro-10-hydroxy-2,3,6-trimethoxydibenz(b,f)oxepin was previously identified in *Dalbergia Stevenson* [19,37]. Pilloin is a flavonoid extracted from the Ovidia pillo-pillo plant, *Marrubium cylleneum*, and propolis.

## 3. Materials and Methods

### 3.1. Wood Samples

**Figure 3:**
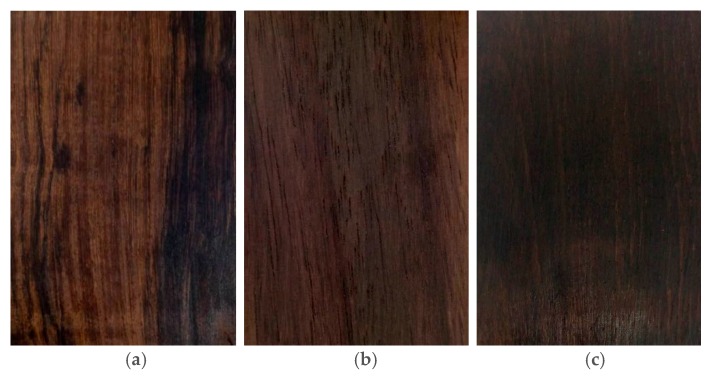
Heartwood of (**a**) Dalbergia cultrate, (**b**) Dalbergia latifolia and (**c**) Dalbergia melanoxylon.

The Latin names, trade names, and places of origin of the *Dalbergia* species are presented in Table 4. The three *Dalbergia* heartwood samples were obtained from the Research Institute of Wood Industry at the Chinese Academy of Forestry, China. Figure 3 shows tangential sections of the three kinds of *Dalbergia* heartwood. Three replicates were analyzed per sample.

### 3.2. Preparation of Wood Extracts

Heartwoods were chopped into thin pieces, air-dried, and finely powdered using an electric grinder (Baijie, Hangzhou, China). Then, 40–60 mesh powders (0.2 g) were extracted in ethanol/water (10 mL, 9:1, *v*/*v*), benzene/ethanol (2:1, *v*/*v*), and ethyl acetate in an ultrasonic bath for 1 h at room temperature. Ethanol (99.8% purity), benzene (99.7% purity), ethyl acetate (99.0% purity), *n*-hexane (98.0% purity) were purchased from Aladdin (Shanghai, China). Next, the mixture was centrifuged for 5 min and filtered through a 0.45-µm pore size filter before the extraction solvent was dried in an oven at 103 ± 2 °C to reach a consistent weight for further analysis.

### 3.3. FTIR Analysis

Two mg of the 90% ethanol extract were mixed with 100 mg of KBr powder in a smooth agate mortar. Then, the mixture was ground and pressed into a transparent pellet. FTIR spectra were obtained within a scanning range of 4000–400 cm^−1^ at a resolution of 4 cm^−1^ and 16 total scans using a Spectrum ONE spectrometer (Perkin Elmer, Waltham, MA, USA) equipped with a DTGS detector at room temperature. The instrument was free of H_2_O and CO_2_.

### 3.4. GC-MS Analysis

GC/MS analysis was carried out by a triple quadrupole GC-MS system 450GC-320MS, (Bruker Billerica, MA, USA). Separation was achieved using a DB-5MS column (30 m × 0.25 mm × 0.25 μm; Agilent Technologies, Santa Clara, CA, USA) with a temperature program from 50 °C (5 min) to 290 °C (12 min) at 10 °C/min with helium as the carrier gas (1 mL/min). The solvent was delayed 4.5 min; the injection volume was 1 μL at a split ratio of 10. The mass spectrometer was operated in electron impact mode (70 eV), and masses were scanned over a range of 40–800 *m*/*z*. The transmission line temperature was 250 °C, and the ion source temperature was 200 °C. Peak assignment was accomplished by comparing the MS spectra to the National Institute of Standards and Technology (NIST 2010) library.

## 4. Conclusions

This study reveals that *Dalbergia cultrate*, *Dalbergia latifolia*, and *Dalbergia melanoxylon* can be successfully distinguished based on extractive analysis. Initially, the extract content of the three species was found to be different, with *Dalbergia melanoxylon* demonstrating the highest extractive content. By comparing different solvents, the extraction components were found to be mostly polar. Then, FTIR spectra effectively revealed additional information about the functional groups of the extractive components. We also identified the unique and primary components using GC-MS. The main chemical components of the three species varied, and 7-methoxy-1-thioflavone, 4,2′,3′,4′- tetramethoxy-5′-methyl-6-methylaminomethyl-1,1′-biphenyl and 10,11-dihydro-10-hydroxy-2,3,6- trimethoxydibenz(b,f)oxepin exhibited the highest relative content of the three species, respectively. Furthermore, each species contained its own characteristic components, a useful finding for distinguishing between the three species. In summary *Dalbergia cultrate*, *Dalbergia latifolia*, and *Dalbergia melanoxylon* can be distinguished successfully according to differences in extractive content, functional groups, and chemical composition.

## Figures and Tables

**Table 1:** Comparison of extractive contents in different solvents.

Species	Yield of Extractives (%)			
	Ethanol/Water (9:1, *v*/*v*)	Benzene/Ethanol (2:1, *v*/*v*)	Ethyl Acetate	*n*-Hexane
*Dalbergia cultrate*	11.6	8.6	6.2	-
*Dalbergia latifolia*	10.1	9.65	6.8	-
*Dalbergia melanoxylon*	16.65	16.05	13.0	-

**Table 2:** FTIR band assignment.

Wavenumbers(cm^−1^)			
*D. Cultrata*	*D. Latifolia*	*D. Melanoxylon*	Band Assignments
3381	3400	3401	O-H stretch; N-H stretch
2932	2933	2935	C-H stretch: CH_2_
2849	2839	2841	C-H stretch:CH_3_
1691	1669	1675	C=O stretch; C=N stretch
1602	1602	1602	C=C stretching of aromatic skeleton
1555	-	-	C=C stretching of aromatic skeleton
1510	1508	1510	C-C stretch bands within ring skeleton
-	1452	-	Skeletal C-C stretching; CH_3_ symmetrical bending vibrations ;CH_2_ scissoring
1445		1444	Aromatic stretching(flavonoids); CH_3_ symmetrical bending vibrations
1378	-	-	CH_3_ asymmetrical bending vibrations
		1366	CH_3_ symmetrical bending; in-plane C-OH bending rical bending
-	1352		CH_3_ symmetrical bending; in-plane C-OH bending C-N-C asymmrtric vibration of aromatic compounds
-	1318	-	in-plane C-OH bending; C-O stretching
1286	-	-	C-O-C stretching (flavonoids)
-	1270	1273	C-O vibration
1203	1200	1199	C-O-C stretching
-	1170	-	C-O stretching C-O-C stretching or frame vibration (flavonoids)
1153	-	-	C-O-C stretching(flavonoids); in-plane C-OH bending C-O-C stretching or frame vibration (flavonoids)
-	-	1130	C-OH stretching
1114	-	-	C-OH stretching
1016	1029	1023	C-O stretching
999	-	-	C-H out-of-plane bend C-O stretching
-	917	-	C-H out-of-plane bend
875	878	874	C-O stretching
-	839	836	C-H stretching out of plane of aromatic ring; C-N wagging
699	700		C-H stretching out of plane of aromatic ring; C-S stretching

**Table 3:** Chemical composition of extractives analyzed by GC/MS.

ID	RT(min)	Compounds	Molecular Structure	Releative Content (%) *		
				*D. Cultrate*	*D. Latifolia*	*D. Melanoxylon*
1	24.97	Phenol,4-methyl-2-[5-(2-thienyl)pyrazol-3-yl]-	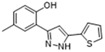	4.76	12.41 (0.7)	1.47
6	29.66	13-Docosenamide, (*Z*)-	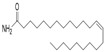	2.08 (0.2)	2.17 (0.2)	1.26 (0.1)
2	25.13	Naphtho[2,3-b]furan-4,9-dione, 2-isopropyl-	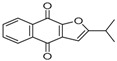	1.54	1.36 (0.2)	-
5	28.90	1-Thioflavone, 7-methoxy-	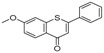	25.55 (0.2)	4.23	-
3	26.44	3,3′,4,4′-Tetramethoxystilbene	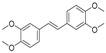	1.49	-	-
4	27.26	3,7,3′,4′-Tetrahydroxyflavone	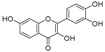	10.78 (0.1)	-	-
7	32.49	Parietin	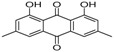	24.81 (0.4)	-	-
8	24.11	1,7,7-Trimethyl-3-phenethylidenebicyclo[2.2.1]heptan-2-one	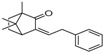	-	3.28	-
9	24.73	Phenol, 4,4′-methylenebis[2,6-dimethyl-	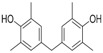	-	1.60	-
10	26.32	1,1′-Biphenyl, 4,2′,3′,4′-tetramethoxy-5′-methyl-6-methylaminomethyl-	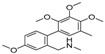	-	29.75 (0.8)	-
11	27.88	(4-Methylsulfanylphenyl)carbamic acid, 2,6-dimethoxyphenyl ester	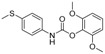	-	1.38	-
12	27.76	10,11-Dihydro-10-hydroxy-2,3- dimethoxydibenz(b,f)oxepin	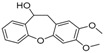	-	-	4.57
13	28.66	2-(4-Methoxy-2,5-dimethyl-phenyl)-9-methyl-2*H*-benzo[g]indazole	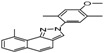	-	-	3.14
14	28.88	10,11-Dihydro-10-hydroxy-2,3,6-trimethoxydibenz(b,f)oxepin	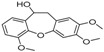	-	-	37.30 (0.8)
15	30.06	10,11-Dihydro-2,3,6-trimethoxydibenz(b,f)oxepin-10-one	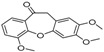	-	-	8.32 (0.2)
16	34.86	Pilloin	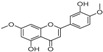	-	-	7.77 (0.1)

* The percentage was calculated based on the peak area. Values in the parentheses are the deviations of three replicates. Deviations lower than 0.1% are not listed in the table.

**Table 4:** Latin and trade names and places of origin of investigated *Dalbergia* species.

Latin Name	Trade Name	Place of Origin
*Dalbergia cultrate* Benth.	Burmese blackwood	Laos
*Dalbergia latifolia* Roxb.	Indian rosewood	Indonesia
*Dalbergia melanoxylon* (Guill. & Perr.)	African blackwood	Mozambique
